# Supplementary material for: In situ‐crosslinked Zippersomes enhance cardiac repair by increasing accumulation and retention
Source: Bioeng Transl Med. 2024 Aug 20;9(6):e10697. doi: 10.1002/btm2.10697 (PMC11558206; doi:10.1002/btm2.10697)
Supplement: Supplementary file 1 — Data S1. Supporting Information. [file BTM2-9-e10697-s001.docx]

# **Supplemental Information**

# **In Situ-Crosslinked Zippersomes Enhance Cardiac Repair by Increasing Accumulation and Retention**

Natalie E. Jasiewicz^1^, Kuo-Ching Mei^1^, Hannah M. Oh^1^, Emily, E. Bonacquisti^1^, Ameya Chaudhari^1^, Camryn Byrum^1^, Brian C. Jensen^2,3^, Juliane Nguyen^1*^

^1^ Division of Pharmacoengineering and Molecular Pharmaceutics, Eshelman School of Pharmacy, University of North Carolina, Chapel Hill, NC 27599, USA

^2^ McAllister Heart Institute, University of North Carolina, Chapel Hill, NC 27599, USA

^3^ Department of Medicine, Division of Cardiology, University of North Carolina,

Chapel Hill, NC 27599, USA

Correspondence to: Juliane Nguyen (julianen@email.unc.edu)

**
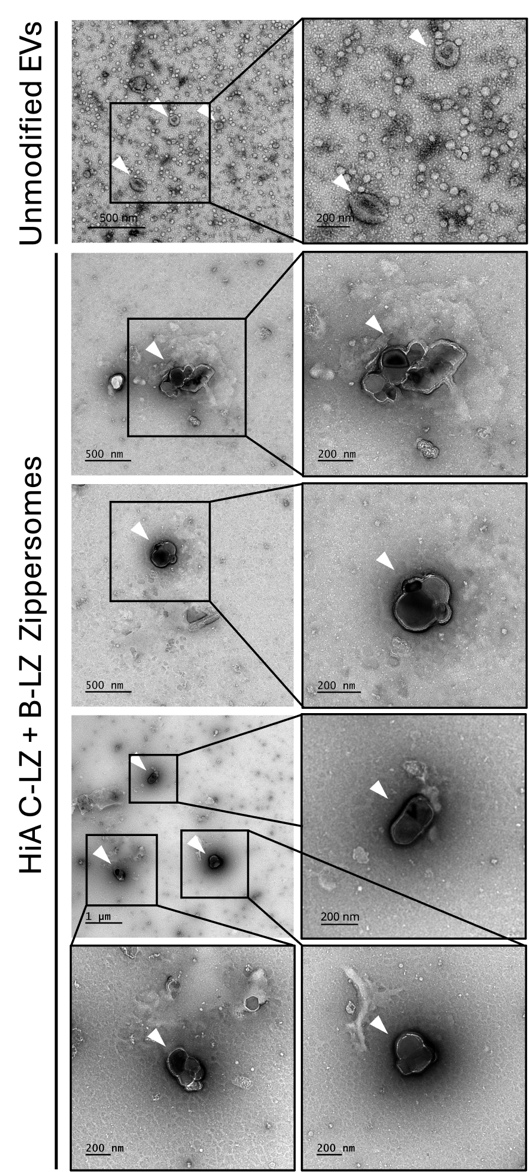
**

**Supplemental Figure 1.** Representative Overview TEM images of unmodified and Zippersome (HiA C-LZ + B-LZ) samples. White arrows indicate EVs and Zippersomes.

**
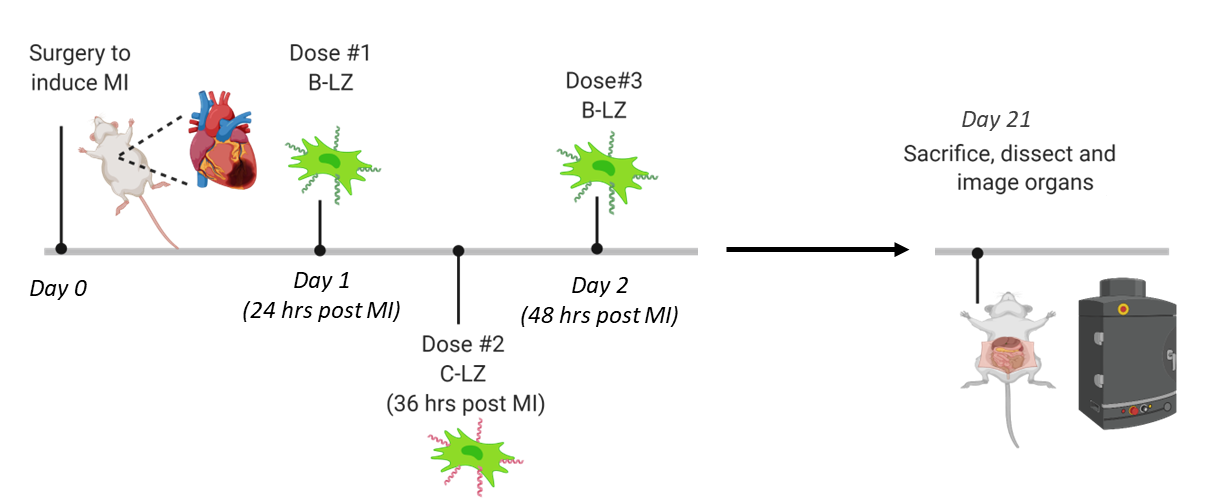
**

**Supplemental Figure 2.** In vivo dosing schedule.

**
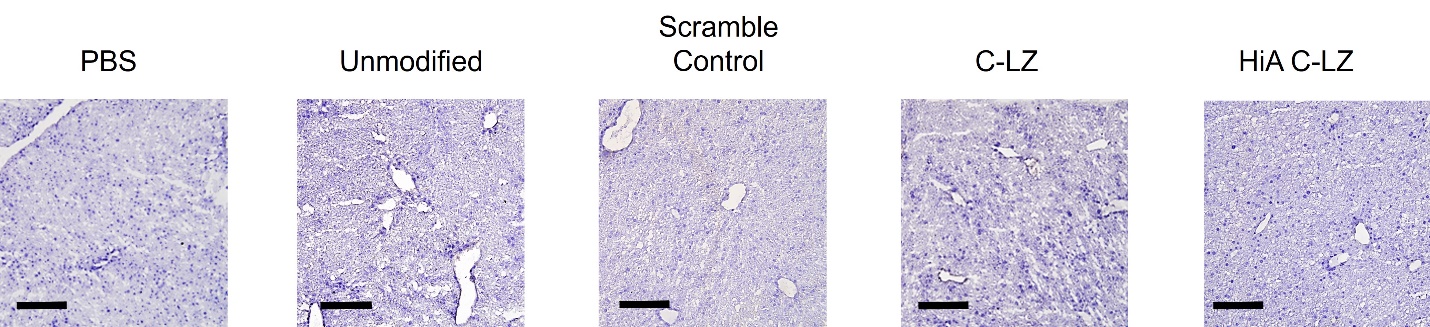
**

**Supplemental Figure 3.** Additional representative CD45 stained liver sections (Day 21). See Figure 5 in the main manuscript for additional images. Scale bar = 200 µm

**
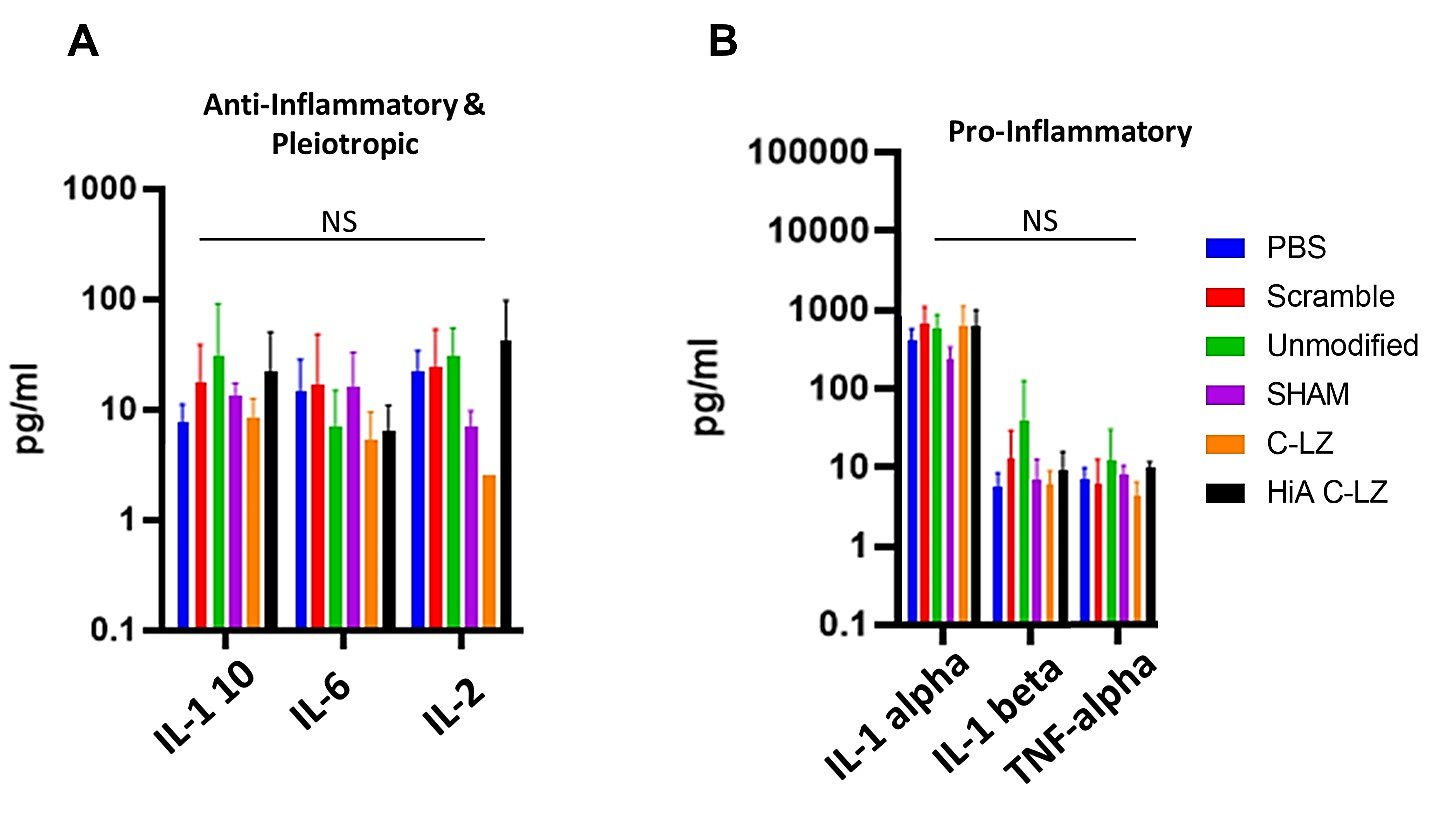
**

**Supplemental Figure 4.** Representative cytokine analysis of Day 21 mouse serum. A) Anti-inflammatory and pleiotropic cytokine expression. B) Pro-inflammatory cytokine expression. Blue = PBS, Red = Scramble Control, Green = Unmodified EV control, Purple = SHAM control, Orange = C-LZ, and Black = HiA C-LZ. The dosing schedule was performed as described above, including a total of 3 injections per mouse. N=10.

| **Ch** | **Antibody** | **Fluorophore** |
| --- | --- | --- |
| 1 | MHCII | biotin (+ Streptavidin-v500) |
| 2 | CD11B | BV605 |
| 3 | CD3 | BUV395 |
| 4 | B220 | spark violet 538 |
| 5 | CD11C | BV480 |
| 6 | CD68 | VioBlue |
| 7 | CD31 | BV421 |
| 8 | THY1 | AF488 |
| 9 | CLEC9A | PE |
| 10 | F4/80 | APC |
| 11 | SIRP | AF594 |
| 12 | FSP | CF633 |

**Supplemental Table 1:** Antibody staining panel for spatial analysis. Antibodies and their conjugated fluorophore used for multiplex spatial analysis of ZipperSome and PBS treated mouse hearts.

**Supplemental Table 2.** Cell type identification markers. Data are presented as mean ± SD with *p <0.05, **p<0.01, ****p<0.0001 by unpaired t test

**Supplemental Figure 5:** **Endothelial cell percentage of total cell count from spatial analysis.** Comparison of percentages of endothelial cells out of total cells detected in PBS-treated mice and HiA-ZipperSome treated mice 21 days after myocardial infarction via spatial analysis. Data are presented as mean ± SD with *p <0.05, **p<0.01, ****p<0.0001 by unpaired t test. N=3

**Supplemental Figure 6:** **M1 macrophage composition**. Comparison of percentages of pro-inflammatory (M1) phenotypic cells out of total detected macrophages within heart samples of PBS treated mice and HiA-ZipperSome treated mice. Data are presented as mean ± SD with *p <0.05, **p<0.01, ****p<0.0001 by unpaired t test. N=3.
